# Relations Between Preferential Looking to Synchronous Audiovisual Speech and Expressive Language in Infants With Autistic and Non‐Autistic Siblings

**DOI:** 10.1111/mbe.70039

**Published:** 2026-02-12

**Authors:** S. Madison Clark, Jacob I. Feldman, Jennifer E. Magnuson, Grace Pulliam, Pooja Santapuram, Sarah Bowman, Catherine T. Bush, Kacie Dunham‐Carr, Sweeya V. Raj, Bahar Keçeli‐Kaysılı, David J. Lewkowicz, Tiffany G. Woynaroski

**Affiliations:** ^1^ Neuroscience Undergraduate Program Vanderbilt University; ^2^ Department of Hearing & Speech Sciences Vanderbilt University Medical Center; ^3^ Frist Center for Autism and Innovation Vanderbilt University; ^4^ Department of Hearing & Speech Sciences Vanderbilt University; ^5^ Neuroscience Graduate Program Vanderbilt University; ^6^ Vanderbilt Brain Institute Vanderbilt University; ^7^ Vanderbilt University School of Medicine; ^8^ Child Study Center, Yale University School of Medicine; ^9^ Department of Psychology Yale University; ^10^ Vanderbilt Kennedy Center Vanderbilt University Medical Center; ^11^ Department of Communication Sciences and Disorders, John A. Burns School of Medicine University of Hawai'i at Mānoa

## Abstract

Differences in audiovisual processing may influence language development in autism. We characterized preferential looking to temporally synchronous audiovisual speech in fifty infants (28 elevated‐likelihood [54% male]; 22 population‐level‐likelihood [50% male]) aged 12–18 months. We first tested whether preferential looking to synchronous audiovisual speech differed between groups; population‐level‐likelihood infants demonstrated greater preferential looking to synchronous audiovisual speech relative to elevated‐likelihood infants by 18 months. We then assessed whether preferential looking to synchronous audiovisual speech was related to concurrent expressive language (1) directly, (2) via vocalization complexity, and (3) as moderated by age, sex, and familial likelihood group. Infants completed an eye tracking task and standardized communication and language assessments at 12–18 months. The population‐level‐likelihood infants demonstrated greater preferential looking to synchronous audiovisual speech relative to elevated‐likelihood infants by 18 months. Preferential looking to synchronous audiovisual speech was not directly and unconditionally associated with concurrent expressive language. However, moderation and mediation analyses showed that preferential looking to synchronous audiovisual speech was significantly associated with concurrent expressive language through increased vocalization complexity in male infants. Implications for research, theory, and clinical practice are discussed.

## LAY ABSTRACT

In infants at elevated‐ and population‐level‐likelihood for autism, we investigated if preferential looking to a synchronous talking face was related to emerging language abilities. Male infants who looked more to the synchronous talking face tended to have more mature vocalizations, which translated to higher expressive language. For male infants in both likelihood groups, less looking to a synchronous talking face early in life may be related to differences in learning how to use spoken language.

Autism is a neurodevelopmental condition characterized by differences in social communication and the presence of restricted, repetitive patterns of behavior, interests, or activities. Differences in sensory functioning have also now been recognized as a part of the diagnostic criteria for autism since the fifth edition of the Diagnostic and Statistical Manual of Mental Disorders (DSM‐5; American Psychiatric Association, 2013, 2022). Though current diagnostic criteria do not consider language differences among the core features of autism, expressive language is highly heterogeneous in autistic individuals (Kjelgaard & Tager‐Flusberg, 2001). While some autistic individuals may not use any expressive language, other autistic individuals may present with average or even above average expressive language abilities (e.g., Bacon, Osuna, Courchesne, & Pierce, 2019; Norrelgen et al., 2015). Importantly, expressive language acquired early in life is the single‐most replicated predictor of long‐term social, educational, and vocational outcomes for autistic individuals (e.g., Billstedt, Gillberg, & Gillberg, 2007; Rutter, Greenfeld, & Lockyer, 1967). Thus, there is a need to identify factors that may explain differences in early expressive language abilities in autism.

## Audiovisual Speech Processing May Explain Differences in Expressive Language in Autism

One factor that may explain differences in early expressive language ability in autism is audiovisual speech processing (e.g., Bahrick & Todd, 2012; Wallace, Woynaroski, & Stevenson, 2020). It has been proposed that early differences in the processing of sensory information, in particular auditory and visual speech information (i.e., the auditory speech signal and the corresponding, temporally synchronized movements of the mouth), may cascade onto differences in language development (Cascio, Woynaroski, Baranek, & Wallace, 2016; Wallace et al., 2020). A growing body of research has provided increasing empirical support for this “cascading effects” theory (Cascio et al., 2016; Robertson & Baron‐Cohen, 2017), demonstrating that processing, perception, and integration of audiovisual stimuli are associated with expressive language ability in autistic children (e.g., Edgar, Todd, & Bahrick, 2022; Foxe et al., 2015; Woynaroski et al., 2013; see Feldman et al., 2018 for a systematic review). The extant literature suggests that associations between audiovisual processing and higher‐order skills such as language are strongest for audiovisual stimuli that are social in nature, such as audiovisual speech, and are likely greater earlier versus later in life (Feldman et al., 2018).

## A Need to Focus on Infant Siblings

Although the literature suggests that links between audiovisual processing and language in autism are more pronounced earlier in life (see Beker, Foxe, & Molholm, 2018 for a review), few studies have evaluated relations between audiovisual speech processing and expressive language in the earliest stages of development (i.e., in infancy; within the window wherein we know that such audiovisual speech processing and language skills are emerging). This gap exists largely because autism is difficult to reliably diagnose early in life, especially in infancy (see Woolfenden, Sarkozy, Ridley, & Williams, 2012). Increasingly, infant siblings of autistic children are being prospectively studied, as these infants are at an elevated familial likelihood of receiving a diagnosis of autism (recurrence rate of 18%–20%; Ozonoff et al., 2024) compared to infants at general population‐level‐likelihood for autism (i.e., infants who have only non‐autistic older siblings; incidence rate of 2.76%; Maenner et al., 2023). Notably, elevated‐likelihood infants are also more likely than population‐level‐likelihood infants to exhibit expressive language delays or Developmental Language Disorder (e.g., Belteki, Lumbreras, Fico, Haman, & Junge, 2022; Marrus et al., 2018). Thus, studying infants at elevated‐likelihood for autism provides a unique opportunity to evaluate emerging differences in audiovisual processing and links with expressive language in autism.

## Audiovisual Speech Processing in Infancy

One of the earliest perceptual abilities contributing to audiovisual speech processing that can be assessed in infants is sensitivity to audiovisual coherence, including the detection of audiovisual temporal synchrony (i.e., the ability to detect events occurring at the same time in multiple sensory modalities; Bahrick & Todd, 2012; Soto‐Faraco, Calabresi, Navarra, Werker, & Lewkowicz, 2012). Audiovisual temporal synchrony (both of speech and non‐speech stimuli) facilitates learning from sensory events in infants' natural environments (see Bahrick, 2010). In infants, audiovisual temporal synchrony detection is typically measured via looking behavior in a preferential‐looking paradigm wherein two visual stimuli are presented (i.e., one synchronous and one asynchronous with the auditory stimulus; e.g., Lozano, Belinchón, & Campos, 2024; Pons, Andreu, Sanz‐Torrent, Buil‐Legaz, & Lewkowicz, 2013; see Cox, Keren‐Portnoy, Roepstorff, & Fusaroli, 2022 for a review). This task assumes that infants who perceive the temporal relation between the visual and auditory stimuli will look at the synchronous stimulus longer than the asynchronous stimulus, though the opposite pattern may also be the case (Bahrick, 2010; Lewkowicz, 2000). Previous research suggests that infants from the general population may be able to detect audiovisual synchrony for non‐speech stimuli as young as 4–6 months old (e.g., Bahrick, 1983; Falck‐Ytter et al., 2018) and for speech stimuli as young as 8 months old (e.g., Pons & Lewkowicz, 2014). Preferential looking to temporal synchrony during fluent audiovisual speech (i.e., continuous, passage‐level speech) appears to be detectable by 12–14 months old (e.g., Lewkowicz, Minar, Tift, & Brandon, 2015; Pons & Lewkowicz, 2014).

## Differences in Preferential Looking to Synchronous Audiovisual Speech in Autistic Children

To date, studies evaluating preferential looking to synchronous audiovisual speech in autistic children have been conducted primarily in preschool‐aged children (e.g., Bebko, Weiss, Demark, & Gomez, 2006; Patten, Labban, Casenhiser, & Cotton, 2016; Patten, Watson, & Baranek, 2014; Righi et al., 2018). These studies illustrated that autistic preschoolers, on average, tend to present with reduced preferences for looking toward synchronous versus asynchronous speech compared to non‐autistic comparison groups. Importantly, this work found that increased preferential looking toward synchronous audiovisual speech is related to increased language abilities in autistic and non‐autistic preschoolers (Patten, Watson, & Baranek, 2014; Righi et al., 2018).

## Preferential Looking to Audiovisual Synchrony in Infant Siblings of Autistic Children

To our knowledge, very limited work has examined sensitivity to temporal synchrony via preferential looking to synchronous speech in elevated‐likelihood infants. The few studies that have been conducted suggest that there may be differences in detection of and looking to audiovisual synchrony that are more pronounced for speech than for non‐speech stimuli in infants at elevated‐likelihood for autism (at 10 months; Falck‐Ytter et al., 2018; between 4 and 24 months; Suri, Whedon, & Lewis, 2023). However, the only prior study that has evaluated preferential looking to continuous audiovisual speech to date failed to detect any differences between elevated‐ and population‐level‐likelihood infants at 4, 8, and 12 months (Lozano et al., 2024). As such, additional research is needed to determine whether elevated‐likelihood infants differ from population‐likelihood infants in their preferential looking to synchrony in continuous audiovisual speech and, if so, when such between‐group differences may emerge.

## Prelinguistic Vocal Complexity as a Potential Mechanism by Which Preferential Looking to Synchronous Audiovisual Speech May Influence Expressive Language

To our knowledge, no prior study has evaluated the mechanisms by which preferential looking to synchronous audiovisual speech may influence expressive language in elevated‐likelihood infants. Santapuram et al. (2022), however, found that another early‐developing skill related to audiovisual speech processing, looking to the mouth of a speaker (e.g., Feng et al., 2021)—which is the source of temporally synchronous auditory and visual information (Chawarska, Lewkowicz, Feiner, Macari, & Vernetti, 2022; Sumby & Pollack, 1954)—explained differences in concurrent expressive language via vocalization complexity in infants at both elevated‐ and population‐level‐likelihood for autism at 6–18 months of age. Vocalization complexity, specifically the ability to produce more adult‐like consonants and canonical syllables, supports the transition in language development from vocal play (onset around 4–6 months) and babbling (onset around 6–10 months) to words (onset around 10–12 months; e.g., Oller, Eilers, Neal, & Schwartz, 1999; Patten et al., 2014; Vihman, 2014). Increased vocalization complexity is consistently associated with increased expressive language in children aged 8–48 months in the literature (see McDaniel, Slabock, & Yoder, 2018 for a review).

Given its importance in supporting language development, it is possible that vocalization complexity may similarly explain the link between preferential looking to synchronous audiovisual speech and expressive language. We specifically hypothesized that infants with more advanced audiovisual multisensory speech perception (as indexed by preferential looking toward synchronous audiovisual speech) would display increased vocalization complexity (as indexed by the number of consonants produced and the extent to which infants use canonical syllables to communicate; i.e., a more proximal skill that leverages audiovisual multisensory perception; Woynaroski et al., 2016) and increased linguistic skills (as indexed by expressive language; i.e., a more distal or “developmentally downstream” skill). However, it is possible that this relation may only hold for a subset of infants in our sample (i.e., the relation may be moderated by factors such as familial likelihood group, chronological age, or sex).

## Associations of Interest May Vary According to Chronological Age, Sibling Group, and Sex

It is important to consider whether the hypothesized cascade may vary according to such participant characteristics. Prior work evaluating preferential looking to synchronous audiovisual speech in infants from the general population showed that preference for synchrony may be consolidated rather late in infancy (i.e., beyond 12–14 months; Lewkowicz et al., 2015). Additionally, previous research found that between‐group differences in other aspects of sensory processing were emerging during the second year of life (i.e., between 12 and 18 months of age; Feldman et al., 2021). Given these findings, as well as the natural maturational processes that occur in audiovisual multisensory development (Soto‐Faraco et al., 2012)—maturation which may be delayed in autism (see Beker et al., 2018 for a review)—we hypothesized that effects of interest in the present study may vary according to chronological age.

In other recent studies, relations of interest with expressive language varied for elevated‐likelihood versus population‐level‐likelihood infants (e.g., Chawarska et al., 2022; Feldman et al., 2024; Markfeld et al., 2025; Suri et al., 2023). As such, we also anticipated that familial likelihood group may moderate the hypothesized relation between early preferential looking to synchronous audiovisual speech and concurrent expressive language through vocalization complexity.

Finally, there is some evidence to suggest that audiovisual integration and patterns of looking to talking faces may differ (1) in males versus females in autistic and non‐autistic children (e.g., Harrop et al., 2019; Ross, Del Bene, Molholm, Frey, & Foxe, 2015), (2) in male versus female infants from the general population (e.g., Lozano et al., 2022), and (3) in infants at elevated‐ and population‐level‐likelihood for autism (e.g., Chawarska, Macari, Powell, DiNicola, & Shic, 2016). Recent work in our laboratory has also found that relations between other looking patterns toward talking faces (i.e., looking to the mouth of a speaker) and language in infant siblings are moderated by biological sex (Dunham‐Carr, 2024). Taken together, Lozano et al. (2025) have suggested that increased attention to the articulators may be a protective factor for autism for females. Thus, we hypothesized that relations between preferential looking to audiovisual speech and vocalization complexity and/or expressive language may also be moderated by sex.

## The Current Study

The purpose of this project was to assess this hypothetical model by which preferential looking to synchronous audiovisual speech influences concurrent expressive language via vocalization complexity, considering participant characteristics that may moderate such relations. Our research questions were:

Does preferential looking to synchronous audiovisual speech differ between elevated‐ and population‐level‐likelihood infants? Are group differences moderated by age and/or sex?

We hypothesized that at least some subset of the population‐level‐likelihood infants would exhibit stronger temporal synchrony detection and thus would look more toward the synchronous talking face; however, we acknowledge the possibility that infants could find the asynchronous talking face novel and thus spend more time looking to that face. We specifically anticipated that this group difference would be moderated by age and/or sex, such that we only see the hypothesized group difference for infants who were older and/or male.

Is there a relation between preferential looking to synchronous audiovisual speech and concurrent expressive language skills?

We hypothesized that greater preferential looking to the synchronous talking face would be related to higher concurrent expressive language in our sample.

Does vocalization complexity mediate the relation between preferential looking to synchronous audiovisual speech and concurrent expressive language skills?

We hypothesized that greater preferential looking to the synchronous talking face would be related to greater concurrent expressive language skills via increased vocalization complexity.

Are the aforementioned relations moderated by (1) age, (2) familial likelihood group, and/or (3) sex?

We hypothesized that the relations described in research questions 2 and 3 would be significantly moderated by age (such that we may only observe the hypothesized relations in older infants), likelihood group (such that we may only observe the hypothesized relations in infants at increased familial likelihood), and/or sex (such that we may only observe the hypothesized relations in males).

## Ethics Approval Statement

All study and recruitment procedures were approved by the Vanderbilt University Medical Center Institutional Review Board. All procedures upheld the principles in the Belmont Report and the Declaration of Helsinki, and complied with all relevant regulatory statutes, including the US Department of Health and Human Services Policy for the Protection of Human Subjects and the Health Insurance Portability and Accountability Act of 1996. Prior to their child's participation in the study, caregivers provided written informed consent, and families received compensation for their participation.

## METHODS

### Participants

Participants were 50 infants—28 elevated‐likelihood infants (15 male, 13 female) and 22 population‐level‐likelihood infants (11 male, 11 female)—from a larger longitudinal study of sensory processing and language development in infant siblings at Vanderbilt University Medical Center (i.e., Feldman et al., 2021, 2024; Santapuram et al., 2022). Participants were recruited from the greater Nashville area via community engagement activities at the Adventire Science Center, emails, flyers, social media, university/hospital advertising, word of mouth, and referrals from local doctors' offices and clinics with access to our recruitment materials. Visits to the laboratory occurred between February 2017 and October 2019.

Inclusion criteria for all infants in this study were: (1) chronological age between 12 and 18 months, (2) a monolingual English‐speaking household, and (3) at least one older biological sibling who was either diagnosed with autism (elevated‐likelihood group) or only older biological siblings who had no autism diagnosis (population‐level‐likelihood group). For elevated‐likelihood infants, the older siblings' autism diagnoses were confirmed by a licensed clinician according to DSM‐V criteria and a research reliable administration of the Autism Diagnostic Observation Schedule ([ADOS]; Lord et al., 2012), either within our laboratory or by record review. For population‐level‐likelihood infants, caregivers completed the Social Communication Questionnaire (Rutter, Bailey, & Lord, 2003) for all older siblings to confirm scores on this screening tool were below the threshold for autism concern. Exclusion criteria for all participants included: (1) adverse neurological history, (2) known genetic condition, and (3) pre‐term birth (gestation <37 weeks). Inclusion and exclusion criteria were confirmed at recruitment (i.e., no participants were excluded from analyses for the above reasons). Groups were matched at the group level on both chronological age and caregiver‐reported sex assigned at birth (see Table 1 for descriptive statistics by familial likelihood group and Table S1 for descriptive statistics by familial likelihood group and sex).

**Table 1:** Selected Participant Characteristics by Familial Likelihood Group

Variables	Elevated‐likelihood (*n* = 28), *M* (*SD*), Min–Max	Population‐level‐likelihood (*n* = 22), *M* (*SD*), Min–Max
Age in months	13.89 (1.95) 11–18	13.95 (2.17) 12–18
MSEL‐ELC^a^	89.58 (13.03) 70–118	100.14 (8.02) 84–121

	*n*	*N*
Caregiver‐reported sex assigned at birth		
Male	15	11
Female	13	11
Race		
White	28	20
Black/African American	0	1
Multiple	0	1
Ethnicity		
Hispanic/Latino	1	1
Not Hispanic/Latino	27	21
Primary caregiver's highest level of education		
High school diploma or GED	2	0
College/Technical (1–2 years)	10	3
College/Technical (3–4 years)	8	7
Graduate/Professional school (1–2 years)	5	5
Graduate/Professional school (3+ years)	3	7

*Note*. Elevated‐likelihood = Infants with at least one autistic older sibling, Population‐level‐likelihood = Infants with only non‐autistic older siblings. MSEL‐ELC = Mullen Scales of Early Learning‐Early Learning Composite (Mullen, 1995). This standardized score is commonly used as a proxy for IQ. Likelihood groups were well‐matched on chronological age and sex at the group level (*p* > .5).

^a^ Groups differed at *p* = .001.

### Procedures

All infants were seen in the laboratory between 12 and 18 months of age for one to three visits (depending on infants' tolerance for data collection procedures and family preferences), wherein preferential looking to synchronous audiovisual speech, vocalization complexity, and expressive language were measured (see Table 2 for a list of constructs and variables relevant to analyses). Participants also completed the Mullen Scales of Early Learning (MSEL; Mullen, 1995), which permitted more comprehensive characterization of the sample.

**Table 2:** Summary of Constructs and Variables Relevant to Analyses

Construct	Variable	Role
Preferential Looking to the Synchronous Talking Face	The proportion of time spent looking to the synchronous talking face relative to the total time spent looking to both faces: $\frac{{\mathrm{AOI}}_{\text{synchronous talking face}}}{{\mathrm{AOI}}_{\text{synchronous talking face}}+{\mathrm{AOI}}_{\text{asynchronous talking face}}}$	Dependent variable (RQ1) Predictor variable (RQ2–4)
Expressive Language	Average *z* scores for: Expressive language age equivalency score from the MSEL Raw number of words a child “understands and says” (expressive vocabulary) from the MCDI Expressive communication age equivalency score from the VABS‐2	Dependent variable (RQ2–4)
Vocalization Complexity	Average *z* scores for: Number of consonants from Wetherby's true consonant inventory list used in communication acts across the CSBS (Wetherby & Prizant, 2002) Proportion of intentional communication acts containing canonical syllables across the CSBS	Putative Mediator (RQ3 and RQ4)
Age in Months	Chronological age at the first study visit calculated from date of birth provided per caregiver report on a demographic survey	Putative Moderator (RQ1 and RQ4)
Familial Likelihood Group	Elevated‐likelihood (i.e., infants with at least one autistic older sibling) vs. population‐level‐likelihood infants (i.e., infants who have only non‐autistic older siblings), operationalized according to screening and diagnostic measures administered for older siblings at study entry (ADOS‐2, SCQ).	Putative Moderator (RQ4)
Sex	Caregiver report of whether infants' sex assigned at birth was male or female	Putative Moderator (RQ4)

*Note*. ADOS‐2 = Autism Diagnostic Observation Schedule, Second Edition (Lord et al., 2012); AOI = area of interest; CSBS = Communication and Symbolic Behavior Scale Developmental Profile—Behavior Sample (Wetherby & Prizant, 2002); MCDI = MacArthur‐Bates Communicative Development Inventories (Fenson, Marchman, Thal, Dale, & Reznick, 2007); MSEL = Mullen Scales of Early Learning (Mullen, 1995); RQ = research question; VABS‐2 = Vineland Adaptive Behavior Scales, Second Edition (Sparrow, Cicchetti, & Bella, 2005); SCQ = Social Communication Questionnaire (Rutter et al., 2003).

### Measure of Preferential Looking to Synchronous Audiovisual Speech

Participants' preferential looking to temporally synchronous audiovisual speech was assessed using a previously developed preferential‐looking paradigm (Pons et al., 2013). Each infant viewed four trials of side‐by‐side videos displaying the same female actress reciting a brief monologue in English using infant‐directed speech, with one side (either the left or the right) offset by 0.666 s. This stimulus onset asynchrony was selected based on prior work indicating that infants were able to detect a 0.666 s audiovisual asynchrony in a habituated speech syllable by 4–10 months (Lewkowicz, 2010) and in continuous audiovisual speech by 8 months of age (Pons & Lewkowicz, 2014, Experiment 1). In the first two trials, the videos were presented without audio to assess for any baseline preferential looking to the face on one side of the screen versus the other. In the third and fourth trials, the audio track was presented synchronously with one of the two talking faces. The presentation side of the synchronous talking face was counterbalanced across trials. The first two (silent) trials were 10,000 ms each, and the second two (target) trials were 20,000 ms each for a full task duration of 60,000 ms. The amount of time that each infant spent looking at each face was recorded using an SMI REDn SensoMotoric Instruments (SMI, Teltow, Germany) eye tracker. Prior to presentation of the aforementioned trials, infants completed a five‐point calibration utilizing a flashing star that moved around the screen. A fixation movie (i.e., an Elmo hand puppet waving a rattle) was also presented before each trial to focus the infant's attention on the center of the screen; once the infants attended to the screen, the trial began.

**Fig. 1:**
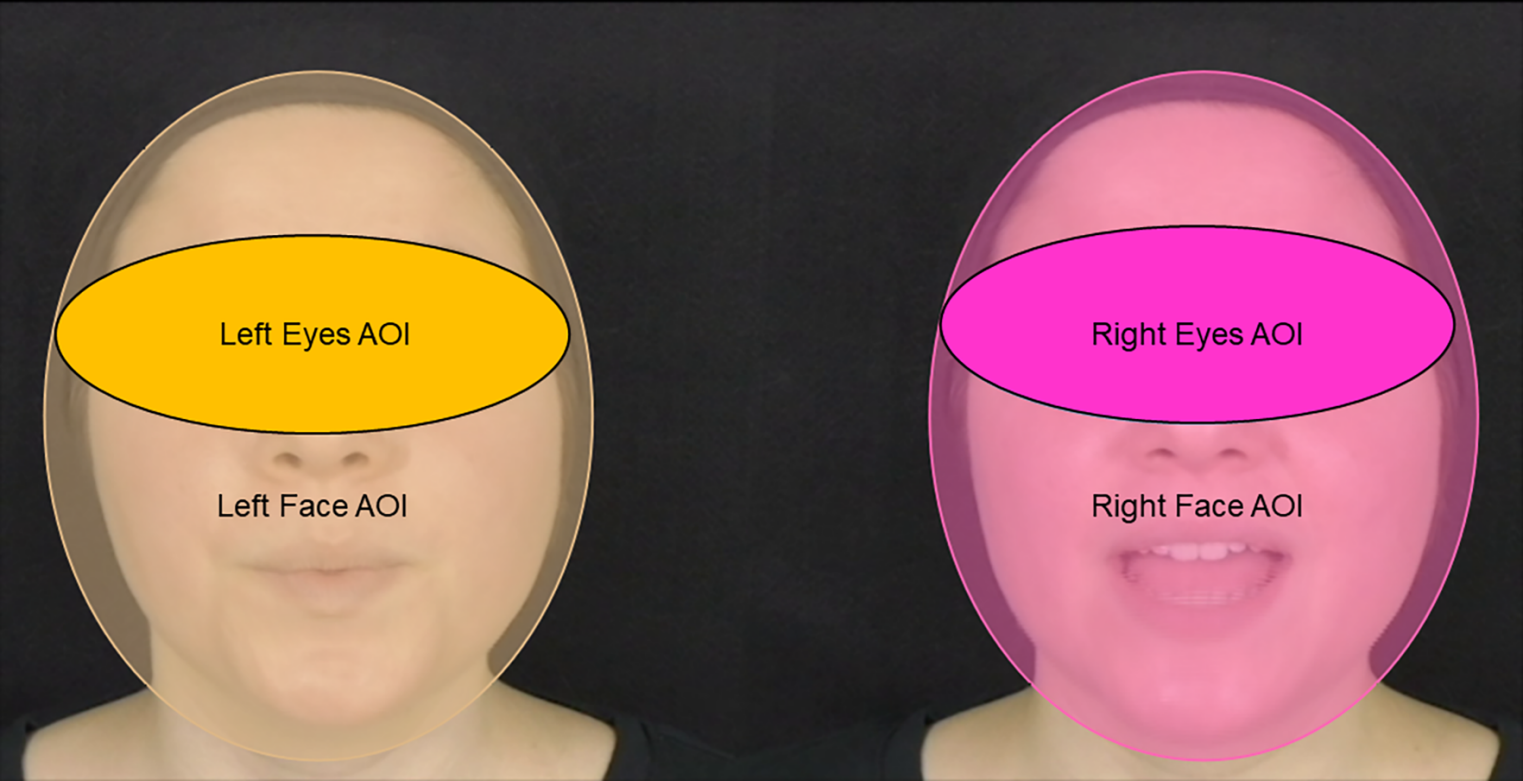
Image of preferential‐looking paradigm with areas of interest (AOIs). Infants completed a task utilizing a preferential‐looking paradigm wherein two talking faces were presented, one on each side of the screen. In the first two trials, the videos were presented without audio. In the third and fourth trials, the audio track was presented synchronously with one of the two faces, while the other face was 0.666 s out of sync. The amount of time infants spent looking within each face area of interest (AOI; *A =* 470,530 pixels each) was recorded via an SMI REDn SensoMotoric Instruments eye tracker. The eye AOIs are depicted in this figure to obscure the identity of the actress in the stimuli and were not utilized in analyses.

To derive our variable indexing preferential looking to the synchronous talking face, the screen was divided into two static areas of interest (AOIs; i.e., one defined around each talking face with an area of 470,530 pixels each), and the duration of fixation for each AOI per trial was quantified via BeGaze analysis software (SensoMotoric Instruments, 2016; see Figure 1). Only trials wherein infants (1) fixated on either face AOI for at least 3,000 ms and (2) fixated on each face AOI for at least 200 ms were retained for analyses; these criteria for trial retention were based on prior studies of audiovisual speech synchrony detection in school‐aged autistic children (e.g., Brown, 2011; Hancock, 2009). Using these criteria, 38 of the 50 participants (22 elevated‐likelihood infants, 16 population‐level‐likelihood infants) yielded usable eye tracking data. According to a chi‐square test of independence, there was no difference in the proportion of usable data across the four trial types (i.e., two silent trials, one with the synchronous talking face on the left, one with the synchronous talking face on the right) according to familial likelihood group, $\chi^{2}$(4) = 2.68, *p* = .613. Additionally, we also found no evidence of bias in looking to one face versus the other during silent trials across groups, *F*(1, 23) = 0.88, *p* = .36, and during all trials across groups *F*(1,21) = 0.17, *p* = .68. Across groups, infants' looking did not significantly differ from chance, *t*(49) = 0.73, *p* = .47, though we could not accept the null hypothesis that looking was equal to chance (see Supporting Information for complete information).

In accord with previous work (e.g., Pons et al., 2013), preferential looking to the synchronous talking face was indexed as the proportion of time that infants spent looking to the synchronous talking face out of the total time spent looking to both faces (i.e., time spent looking to the synchronous talking face/[time spent looking to the synchronous talking face + time spent looking to the asynchronous talking face]).

### Measure of Vocalization Complexity

Infants' vocalization complexity was measured in the context of the Communication and Symbolic Behavior Scales Developmental Profile—Behavior Sample (CSBS; Wetherby & Prizant, 2002), an approximately 30‐min standardized assessment intended to characterize early prelinguistic and linguistic development in children 6–24 months of age. Video‐recorded CSBS samples were coded for intentional communication acts via a 5‐s partial interval coding system, as in Santapuram et al. (2022). Intentional communication acts were defined as: (1) unconventional gestures or nonword vocalizations (e.g., clapping, moving an object toward an adult) with coordinated attention to an object and adult; (2) conventional gestures (e.g., head shake, wave) with attention to an adult; and (3) symbolic forms, including nonimitative words or signs.

For each interval in which an intentional communication act was coded, the interval was then further coded for the presence/absence of vocalizations including canonical syllables and the consonants the infant produced. The number of different consonants produced and the proportion of intentional communication acts that contained canonical syllables (canonical syllabic communication) over the CSBS assessment were derived as indices of vocalization complexity. The number of different consonants produced during communication acts was coded according to Wetherby's True Consonant Inventory List, which includes consonants that emerge early in development, are relatively frequent, and are easy to code reliably (Wetherby & Prizant, 2002). Canonical syllables were operationalized according to: (1) the presence of at least one consonantal sound, (2) the presence of at least one full vowel, and (3) a quick and uninterrupted transition from consonant to vowel or vice versa (Woynaroski et al., 2016).

To be considered reliable in this coding scheme, a coder had to code three consecutive CSBS samples at or above 80% reliability with previously established consensus codes for each sample. Following initial training to this reliability criterion, 20% of CSBS samples were randomly selected and independently coded by a secondary coder to monitor for interrater reliability over the course of coding and to reduce the likelihood of coder drift. Interrater reliability quantified using ICCs was .90 for canonical syllabic communication and .75 for consonant inventory (reflecting excellent and good reliability, respectively).

Consistent with prior work (Santapuram et al., 2022; Woynaroski et al., 2016), (1) the number of different consonants produced from Wetherby's true consonant inventory list and (2) the proportion of intentional communication acts that contained canonical syllables were averaged to create one aggregate *z*‐score indexing vocalization complexity (see Supporting Information Methods and Table S2).

### Measures of Expressive Language

We generated an expressive language aggregate by averaging *z*‐scores for: (1) age equivalency scores for expressive language from the Mullen Scales of Early Learning (MSEL; Mullen, 1995), (2) age equivalency scores for expressive communication from the Vineland Adaptive Behavior Scales, Second Edition (VABS‐2; Sparrow et al., 2005), and (3) the number of words that caregivers reported that their child “understood and said” on the MacArthur‐Bates Communicative Development Inventories: Words and Gestures (MCDI; Fenson et al., 2007; see Supporting Information Methods and Table S2).

### Analytic Plan

All variables used in primary analyses were checked for skewness >|1.0| and kurtosis >|3.0|. One component variable for expressive language (the MCDI expressive vocabulary score) was positively skewed but was corrected with a square root transformation. We utilized the *missForest* package in R (R Core Team, 2023) to impute discrete missing data (Stekhoven & Bühlmann, 2012).

To address our first research question (RQ1), an independent samples *t*‐test was performed to evaluate whether there were between‐group differences in preferential looking to the synchronous talking face between elevated‐likelihood and population‐level‐likelihood infants. A moderation analysis was conducted in PROCESS to assess whether group differences were moderated by chronological age and/or sex (Hayes, 2022).

To test our second research question (RQ2), a regression analysis was used to test the hypothesized (total) relation between preferential looking to the synchronous talking face and concurrent expressive language. To address our third research question (RQ3), a mediation model was carried out in PROCESS to evaluate whether the relation between preferential looking to the synchronous talking face and concurrent expressive language was mediated by vocalization complexity.

To answer our fourth research question (RQ4), additional models were run in PROCESS to evaluate whether any of the paths comprising the direct and indirect effect of preferential looking to the synchronous talking face on concurrent expressive language via vocalization complexity were moderated by (1) age, (2) familial likelihood group, or (3) sex. Moderators were retained for paths on which they accounted for significant variance. To interpret significant moderation models involving the continuous moderator of age (i.e., regression models wherein the interaction term involving age was significant), Johnson‐Neyman tests were utilized to identify regions of significance (i.e., the values of the moderator variable age where the relation between the predictor and dependent variable was significant; Hayes, 2022).

## RESULTS

### Between‐Group Differences in Preferential Looking to Synchronous Audiovisual Speech

**Fig. 2:**
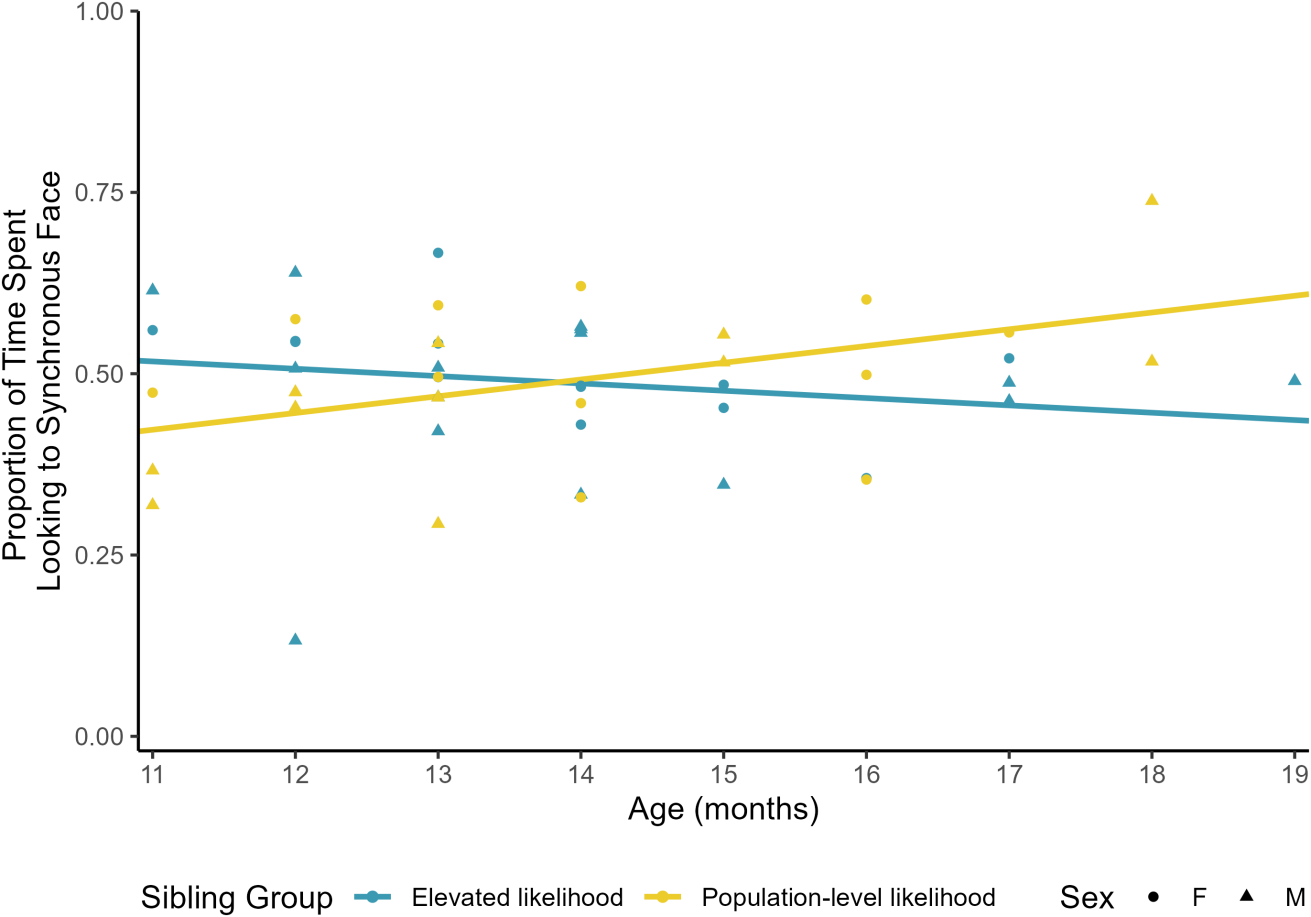
Differences in looking to the synchronous talking face are moderated by chronological age. Elevated‐likelihood = Infants with at least one autistic older sibling, Population‐level‐likelihood = Infants with only non‐autistic older siblings. The relation between looking to the synchronous talking face (*y*‐axis) and group (line color) was significantly moderated by chronological age (*x*‐axis). Sibling groups diverged over the 12–18‐month window, with population‐level‐likelihood infants demonstrating significantly greater preferential looking to synchronous audiovisual speech, on average, relative to elevated‐likelihood infants by 18 months of age. This relation was not moderated by sex.

An independent samples *t*‐test indicated no significant difference between elevated‐likelihood and population‐level‐likelihood infants, on average, in preferential looking to the synchronous talking face, *t*(48) = −0.11, *p* = .914. However, the planned moderation analysis indicated that the between‐group difference in preferential looking to the synchronous talking face was significantly moderated by age, with groups diverging in the preferential looking to synchrony over the 12–18‐month window (*p* value for sibling group × age product term in the regression model testing the moderated effect = .027). A Johnson‐Neyman test probing this significant interaction indicated that population‐level‐likelihood infants displayed significantly greater preferential looking toward the synchronous talking face than elevated‐likelihood infants by 18 months of age (i.e., >17.35 months; see Figure 2). Group differences were not moderated by sex (*p* value for sibling group × sex product term = .963).

### Links With Expressive Language

Regression analyses indicated that preferential looking to the synchronous talking face was not unconditionally related to expressive language (zero‐order correlation = .04; *p* = .772). The total relation between synchrony preference and expressive language was also not significantly moderated by age (*p* value for synchrony preference × age interaction term in regression model testing moderated effect = .093), familial likelihood group (*p* value for interaction term = .069), or sex (*p* value for interaction term = .619).

### Mediation by Vocalization Complexity

**Fig. 3:**
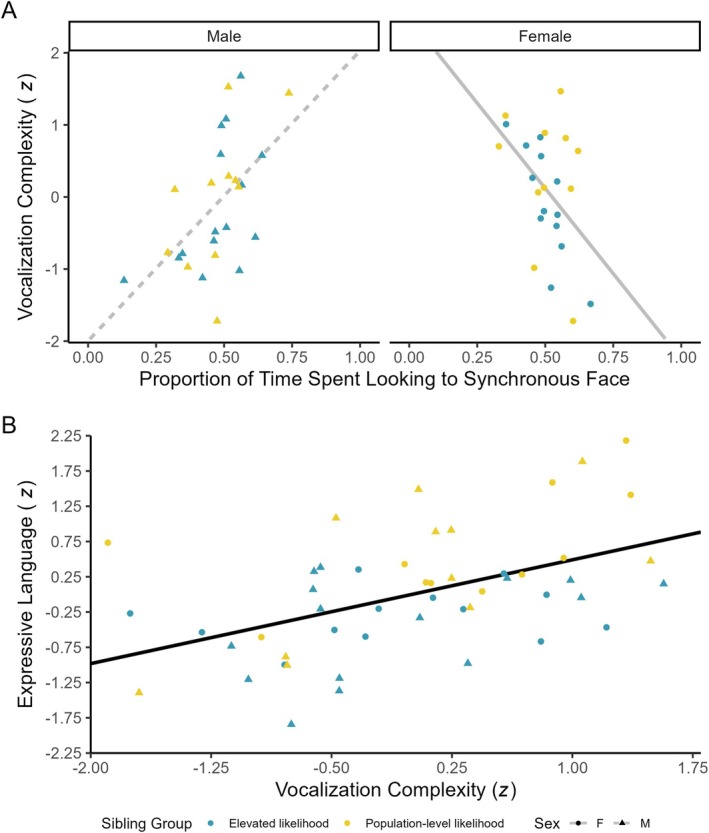
Scatterplots depicting relations relevant to the final mediation model. (a) The relation between preferential looking to synchronous audiovisual speech and vocalization complexity was significantly moderated by sex (*p* for preferential looking to audiovisual speech * sex term in multiple regression model <.001), such that the relation was significant and positive in males (dashed gray line; left panel) and significant and negative in females (solid gray line; right panel). (b) Vocalization complexity was significantly and unconditionally associated with expressive language when controlling for preferential looking to audiovisual speech (*β* = 0.50, *p* < .001; solid black line). Blue dots represent elevated‐likelihood infants, yellow dots represent population‐level‐likelihood infants, circles represent females, and triangles represent males.

**Fig. 4:**
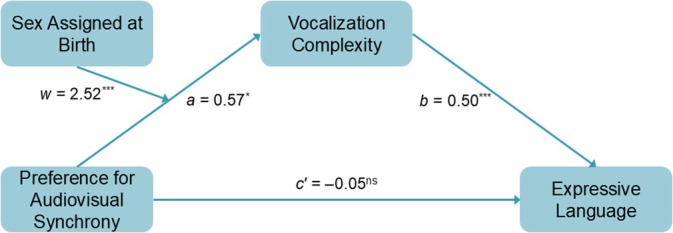
Depiction of significant moderated mediation model. The indirect effect of preferential looking to synchronous audiovisual speech on expressive language via vocalization complexity is reported. The indirect effect varied according to sex. All values are standardized coefficients. *a* = the relation between preferential looking to synchronous audiovisual speech and vocalization complexity, controlling for sex and the interaction between preferential looking and sex; *w* = the interaction term (i.e., the product of sex and preferential looking to synchronous audiovisual speech); *b* = the relation between vocalization complexity and expressive language, controlling for preferential looking to synchronous audiovisual speech; *c'* = the direct effect of preferential looking to synchronous audiovisual speech on expressive communication (i.e., the relation when controlling for vocalization complexity). *p < .05, ***p < .001, ns = nonsignificant result.

In the process of building our mediation model, we found that sex significantly moderated the relation between looking to the synchronous talking face and vocalization complexity (i.e., the *a* path in our statistical model; *p* value for interaction term <.001). Greater looking to the synchronous talking face was significantly associated with increased vocalization complexity in males (*r* = .539, *p* = .005) but was significantly associated with decreased vocalization complexity in females (*r* = −.464, *p* = .023; see Figure 3a). Consequently, we retained sex as a moderator on the *a* path of our mediation model (i.e., using Model 7 in PROCESS; see Figure 4).

The relation between vocalization complexity and expressive language, controlling for preferential looking to the synchronous talking face (i.e., the *b* path in our statistical model) was also statistically significant (*β* = .50, *p* < .001; see Figure 3b). This effect was moderate in magnitude (Cohen's *f* ^2^ = .33) and did not vary according to age, familial likelihood group, or sex (*p* values for product terms in regression models testing moderated effect >.05).

The indirect effect of preferential looking to the synchronous talking face on expressive language through vocalization complexity was significantly moderated by sex (index of moderated mediation = 4.30, 95% CI = [1.79, 7.13]). This indirect effect was significant and positive in males (95% CI for indirect effect = [0.75, 3.74]) but was significant and negative in females (95% CI for indirect effect = [−4.18, −0.31]). Table 3 summarizes the results of the final regressions relevant to this moderated mediation relation. This conditional indirect effect can be interpreted to mean that vocalization complexity explains, at least in part, the relation between preferential looking to the synchronous talking face and expressive language, though it does so in opposite ways for males and females.

**Table 3:** Multiple Regression Results for Final Mediation Model

Model/Variable	*B* (SE)	*β*	*t*	*p*	*f* ^2^
Model 1: Vocalization Complexity (*a* path)					
Constant	2.48 (1.00)	—	2.48	.017	—
2 Looking to AV Synchrony	−4.73 (1.95)	−0.57	−2.42	.020^a^	.127
3 Sex	−4.48 (1.18)	−2.55	−3.81	<.001^a^	.315
4 Interaction	8.76 (2.33)	2.52	3.76	<.001	.307
Model 2: Expressive Language (*b* path)					
Constant	0.19 (0.52)	—	0.38	.706	—
2 Looking to AV Synchrony	−0.41 (1.05)	−0.05	−0.39	.700	.003
3 Vocalization Complexity	0.49 (0.13)	0.50	3.91	<.001	.326

*Note*. Cohen's *f* ^2^ values between .01 and .10 can be interpreted as a small association, and values between .10 and .33 can be interpreted as a medium association (Cohen, 1988). AV = audiovisual; *f* ^2^ = Cohen's *f* ^2^.

^a^ These statistically significant regressor variables are not relevant to the moderation analyses (see Baron & Kenny, 1986).

## DISCUSSION

This study evaluated whether preferential looking to temporally synchronous audiovisual speech differed between infants at elevated‐ and population‐level‐likelihood for autism, and if preferential looking to synchronous audiovisual speech was associated with concurrent expressive language via effects on vocal complexity in these populations. For our first research question, we tested whether preferential looking to synchronous audiovisual speech differed between likelihood groups, and considered whether that group difference was moderated by chronological age and/or sex. While these two groups did not differ in their preferential looking to the synchronous talking face, on average, group differences were significantly moderated by age, such that population‐level‐likelihood infants tended to display greater preferential looking to the synchronous talking face than elevated‐likelihood infants by 18 months of age.

Our second research question assessed whether preferential looking to synchronous audiovisual speech was related to concurrent expressive language skills. We found that there was no unconditional association between preferential looking to the synchronous talking face and concurrent expressive language skills. However, our third and fourth research questions evaluated whether vocalization complexity mediated the relation between preferential looking to synchronous audiovisual speech and concurrent expressive language skills, and whether this model was moderated by (1) age, (2) likelihood group, and (3) sex. We found that there was a significant conditional indirect effect, such that greater looking to the synchronous talking face was associated with increased expressive language skills via increased vocalization complexity in males; this relation was negative in females.

### Group Differences in Preference for Audiovisual Synchrony Are Moderated by Age

While we did not find evidence for unconditional between‐group differences in preference for audiovisual synchrony, the moderation analysis did provide support for a difference between sibling groups that varied according to chronological age. Specifically, our results indicate that likelihood groups diverge in their looking behavior over the 12–18‐month timeframe, with population‐level‐likelihood infants demonstrating significantly greater preferential looking to the synchronous talking face than elevated‐likelihood infants by 18 months of age. Previous work from our lab has demonstrated similar conditional associations regarding sensory differences, specifically suggesting that elevated‐likelihood infants tend to show different patterns of sensory responsiveness toward the end of the 12–18‐month developmental window studied here (Feldman et al., 2021). Together, these findings provide mounting evidence that several key sensory and multisensory differences between these familial likelihood groups are emerging between 12 and 18 months of age.

### Relations between Preference for Synchronous Audiovisual Speech and Expressive Language Are Explained at Least in Part by Vocalization Complexity

The results of mediation analyses indicate that preferential looking to the synchronous talking face is indirectly related to expressive language through its association with vocalization complexity, though this model is moderated by sex, resulting in a significant and positive indirect effect for males and a significant and negative indirect effect for females. This mediated relation is somewhat consistent with prior findings from our team as reported in Santapuram et al. (2022), which found evidence for an indirect link between preferential looking to the mouth of a speaker (the source of the visual speech cues that correspond with the auditory speech signal) and expressive language as mediated by vocalization complexity, though this effect did not vary according to sex. Taken together, these findings support the idea that vocalization complexity may serve as a key mechanism through which infants' early looking to audiovisual speech influences their expressive language development.

It is somewhat surprising that increased looking to a synchronous talking face would be associated with increased expressive language in males but decreased expressive language in females. It could be that our relatively broad age range (i.e., 12–18 months ±30 days) captures a period wherein males are still leveraging the perceptual benefits afforded by looking to multisensory synchrony to build a prelinguistic foundation for future language acquisition while females, with their well‐documented precocious language abilities in this age range (e.g., Eriksson et al., 2012), have surpassed this stage of development. Specifically, at this age females may no longer be relying on their audiovisual processing skills to increase their “more mature” expressive communication (this developmental window may have occurred earlier in life for females). Males, on the other hand, may still be relying on audiovisual redundancy to support their prelinguistic and linguistic communication.

It is notable that the mediation model was moderated by sex but not by familial likelihood group. Given that there are notable sex differences in both language learning trajectories (e.g., Dillon et al., 2023; Harrop et al., 2021) and detection of audiovisual speech coherence (e.g., Lozano et al., 2025) in autistic males versus autistic females, it is possible that the relation between looking to a synchronous talking face and expressive language via vocalization complexity is moderated by both sex and likelihood group; however, we were underpowered to detect this type of “moderated mediation” model (Hayes, 2022). Thus, while we are among the first to contribute to a finer‐grained view of how individual differences in looking to a synchronous talking face are linked with language development and sex in infants, larger scale, longitudinal studies of male and female infants across likelihood groups are necessary to test these hypotheses.

### Strengths, Limitations, and Future Directions

This work represents an important step in understanding the relation between audiovisual temporal synchrony detection in infants at elevated‐likelihood for autism and concurrent language development. Specifically, this study is among the first to evaluate preferential looking to synchronous audiovisual speech in elevated‐likelihood infants relative to population‐level‐likelihood infants. It is also the first investigation to find links between preferential looking to a synchronous talking face and language abilities through vocalization complexity in these two groups of infant siblings.

Crucially, the findings from the present study are constrained by several limitations. First, the current findings are correlational in nature and, as a result, cannot be used to infer causality or directionality. Specifically, even though these findings provide preliminary support for a mediated relation between preference for synchronous audiovisual speech and expressive language, additional research is needed to determine whether the emergence of such a preference early in life is causally related to differences in expressive language through vocalization complexity. As a first step, studies will need to explore longitudinal relations between early preference for synchronous audiovisual speech and later language. Additional work would be needed to evaluate theoretical associations between different aspects of audiovisual speech coherence (i.e., looking to the mouth of a talking face, preference for a synchronous talking face). Such work would be necessary to devise an experimental study that would permit a more rigorous test of the potentially causal link between early looking behavior and expressive language, as the degree of synchrony between the auditory and visual cues in the natural environment cannot be easily manipulated. For example, it has been hypothesized that interventions that encourage infants to look toward the mouth of speakers (i.e., the source of audiovisual redundancy, which may index increased preference for audiovisual speech coherence) could potentially support expressive language development in infants at elevated‐likelihood for autism by boosting prelinguistic skill acquisition (e.g., Tenenbaum, Amso, Abar, & Sheinkopf, 2014; Tenenbaum, Amso, Righi, & Sheinkopf, 2017). If this hypothesis were borne out, there would be increased evidence for causal links between early looking patterns that indicate preference for audiovisual speech coherence and later language. Future work may also assess the link between looking to the mouth and preferential looking to synchronous audiovisual speech; for example, it is possible that looking to the mouth emerges before, and thus facilitates, preferential looking to synchronous audiovisual speech, causing the developmental cascade observed here. Alternatively, preference for a synchronous talking face may be entirely driven by attentional processes to the mouth, which then would cause looking to the mouth to mediate the link between preferential looking to synchronous audiovisual speech and language.

Second, only one stimulus onset asynchrony was assessed in our study design. Future work should include multiple stimulus onset asynchronies (e.g., of differing magnitudes, both audio‐ and visual‐leading; Pons et al., 2013). Doing so will facilitate better characterization of infants' early processing of temporal synchrony in audiovisual speech (e.g., permit an estimation of temporal binding windows in infants), and more thorough evaluation of links between audiovisual synchrony and theoretical constructs of interest (i.e., expressive language). Additionally, future work should explore differences in preference for audiovisual synchrony utilizing non‐speech stimuli in infants at elevated‐likelihood for autism, as several studies have found mixed evidence for significant differences in audiovisual processing for non‐speech stimuli in autistic children (e.g., Bebko et al., 2006; Todd & Bahrick, 2023).

Lastly, our study is limited by our rather homogenous sample. Across sibling groups, the majority of participants were White, non‐Hispanic infants with highly educated caregivers. This lack of diversity limits the generalizability of our findings to the broader population of infant siblings. Future studies should seek to purposefully recruit infants from more diverse backgrounds to better represent the target populations. This is a specific recruitment priority of the ongoing longitudinal study from which these data were drawn, with the goal of achieving greater confidence in the external validity of our findings in future work.

## CONCLUSION

The results of this study provide preliminary empirical support for the hypothesis that preferential looking to synchronous audiovisual speech, as indexed by greater looking to a synchronous talking face, indirectly influences expressive language via vocalization complexity. Our data indicate that infants at population‐level‐likelihood for autism demonstrate greater preferential looking to a synchronous talking face by approximately 18 months of age. Additionally, greater preference for a synchronous talking face is associated with greater expressive language via increased vocalization complexity in male infants. These results provide additional evidence that differences in multisensory processing of audiovisual speech between elevated‐likelihood infants and population‐level‐likelihood infants are present at the later end of the 12–18‐month developmental window and may cascade onto language development. Future work is needed to evaluate whether the relation between looking patterns to speech and expressive language acquisition is causal in nature and to better understand how sex influences such associations.

## CONFLICT OF INTEREST

S. Madison Clark, Catherine T. Bush, Bahar Keçeli‐Kaysılı, Jacob I. Feldman, and Tiffany G. Woynaroski are employed by the Department of Hearing and Speech Sciences at Vanderbilt University Medical Center, which offers communication assessment and intervention services for autistic children through their outpatient clinics and trains clinical students in the provision of assessments and treatments delivered over the course of early childhood. Jacob I. Feldman and Tiffany G. Woynaroski are parents of autistic children. All other authors have no conflicts of interest to declare.

## Supporting information


**Data S1.** Supporting Information.

## Data Availability

All data and analytic scripts are available at https://osf.io/24a89/overview?view_only=c15f0c742651470f9b4b08d7b5317a6d. Data will be uploaded to NDAR at the conclusion of the parent project.
