# Supplementary material for: Relations Between Preferential Looking to Synchronous Audiovisual Speech and Expressive Language in Infants With Autistic and Non‐Autistic Siblings
Source: Mind Brain Educ. 2026 Feb 12;20(1):e70039. doi: 10.1111/mbe.70039 (PMC13097087; doi:10.1111/mbe.70039)
Supplement: Supplementary file 1 — Data S1. Supporting Information. [file MBE-20-e70039-s001.docx]

**Relations Between Preferential Looking to Synchronous Audiovisual Speech and Expressive Language in Infants with Autistic and Non-autistic Siblings**

**Supplemental Material**

S. Madison Clark*, Jacob I. Feldman*, Jennifer E. Magnuson, Grace Pulliam,
Pooja Santapuram, Sarah Bowman, Catherine T. Bush, Kacie Dunham-Carr,
Sweeya V. Raj, Bahar Keçeli-Kaysılı, David J. Lewkowicz,
& Tiffany G. Woynaroski

* Authors made equal contributions

**Supplemental Methods**

**Participants**

For a summary of selected variables by familial likelihood group and sex, see Table S1.

**Intercorrelation of Proposed Component Variables and Generation of Aggregates**

Aggregate scores were calculated by averaging *z*-scores, which were computed using the means and standard deviations for the full sample (consistent with prior work; e.g., Feldman et al., 2021, 2024; Markfeld et al., 2023; Santapuram et al., 2022), and were utilized in analyses to increase the stability, and thus the potential construct validity, of our scores (Rushton et al., 1983) and to reduce the number of analyses.

A priori, we specified that component variables purported to tap the same construct had to be correlated at *r* ≥ .5 to be considered sufficiently intercorrelated to warrant aggregation. All component variables purported to tap expressive language were sufficiently intercorrelated to support aggregation (*r* values ≥ .55, *p* values < .001; see Table S2). Similarly, the component variables for vocalization complexity (consonant inventory and proportion of intentional communication acts containing canonical syllables) were also highly intercorrelated (*r* = .57; *p* < .001), supporting the derivation of a vocalization complexity aggregate from these variables following *z*-score transformation. Importantly, the variables that composed the expressive language aggregate were not correlated with the variables that comprised the vocalization complexity aggregate above the a priori threshold of *r* ≥ .5 (see Table S2).

**Supplemental Analyses**

**Evaluation of Bias in Looking Behavior**

***In Silence***

As a preliminary check on the validity of our data, we conducted a 2 (silent trial; i.e., trial 1 versus trial 2) x 2 (side; i.e., right face versus left face) ANOVA to evaluate whether infants displayed preferential looking in silence. There was no main effect of side, *F*(1,23) = 0.88, *p* = .36, suggesting that infants did not prefer looking to the face on one side of the screen versus the other in silence. The interaction effect was also not significant, *F*(1,23) = 1.29, *p* = .27, indicating that looking preference between faces on the screen did not differ between the two silent trials. Thus, there was no evidence that infants presented with bias in looking behavior toward the face on one side of the screen versus the other in the silent trials.

***All Trials***

We additionally conducted a 4 (trial; silent trial 1, silent trial 2, test trial 1, test trial 2) x 2 (side; i.e., right face, left face) ANOVA to evaluate whether preferences towards one face was present throughout all trial types. There was no main effect of side, *F*(1,21) = 0.17, *p* = .68, suggesting that infants did not prefer looking to the face on one side of the screen versus the other across all trials. The interaction effect was again not significant, *F*(2,45) = 0.19, *p* = .84, indicating that looking preference between faces on the screen did not differ between the two silent trials (note that a Greenhouse-Geisser correction was applied to this factor due to a violation of the assumption of sphericity). Thus, there was no evidence that infants presented with bias in looking behavior toward the face on one side of the screen versus the other across all trial types.

**Differences in Total Looking to Either Face**

As an additional preliminary check on the validity of our data, we assessed whether groups differed in their looking to both faces (i.e., the denominator in our preferential looking to audiovisual synchrony variable) during the test trials. The groups did not differ in their total time spent looking to the face AOIs in the test trials, *t*(13) = 0.82, *p* = .43.

**Evaluation of Null Hypothesis that Looking is Equal to Chance**

As an additional preliminary check, we sought to test the null hypothesis that preferential looking to the synchronous face is equal to chance performance (i.e., µ = .5), with the alternative hypothesis that preferential looking to the synchronous face differs from chance (i.e., µ ≠ .5). We first tested the null hypothesis using one-sample *t*-tests; results indicated that preference for audiovisual synchrony did not significantly differ from chance across groups (*t*(49) = 0.73, *p* = .47), nor within groups for elevated-likelihood (*t*(27) = 0.62, *p* = .54) and population-level-likelihood infants (*t*(21) = 0.39, *p*= .70).

Given that these tests did not indicate significant difference from chance performance, we additionally utilized a Test for Practical Equivalence using the *bayestestR* package in R. Using a Region of Practical Equivalence with an upper-bound of .6 and a lower-bound of .4, we found that the null hypothesis that looking differed from chance could neither be accepted nor rejected across groups (95% Highest Density Interval [HDI] = .[30, .66]), for elevated-likelihood infants (95% HDI = [.27, .65]), and for population-level-likelihood infants (95% HDI = [.31, .68]).

Taken together, while we cannot conclude that looking to the synchronous face does not differ from chance, we also cannot conclude that looking to the synchronous face reflects chance performance, on average, for infants in our sample. Rather, there is substantial heterogeneity that is appropriate to explore.

**Table S1**

*Selected Participant Characteristics by Familial Likelihood Group and Sex*

|  | Elevated likelihood | | |  | Population-level likelihood | | |
| --- | --- | --- | --- | --- | --- | --- | --- |
| Variable | Total  (*n* = 28)  *M* (*SD*) | Males  (*n* = 15)  *M* (*SD*) | Females  (*n* = 13)  *M* (*SD*) |  | Total  (*n* = 22)  *M* (*SD*) | Males  (*n* = 11)  *M* (*SD*) | Females  (*n* = 11)  *M* (*SD*) |
| Age in Months | 13.89 (1.95) | 14.00 (2.20) | 13.77 (1.69) |  | 13.95 (2.17) | 13.73 (2.49) | 14.18 (1.89) |
| Looking to AV  Synchrony | .49 (.11) | .47 (.13) | .50 (.07) |  | .49 (.11) | .48 (.12) | .50 (.10) |
| MSEL-ELC | 89.58 (13.03) | 90.99 (12.25) | 88.00 (14.21) |  | 100.14 (8.02) | 99.91 (4.97) | 100.36 (10.50) |
| VABS Expressive Communication AEQ | 12.11 (3.64) | 11.80 (4.13) | 12.46 (3.13) |  | 16.00 (3.16) | 15.27 (3.68) | 16.73 (2.49) |
| MCDI Expressive Vocabulary | 8.04 (7.03) | 8.53 (8.31) | 7.46 (5.46) |  | 22.23 (18.28) | 18.27 (18.26) | 26.18 (18.28) |
| MSEL Expressive Language AEQ | 11.94 (2.36) | 11.55 (2.70) | 12.38 (1.89) |  | 13.95 (3.54) | 14.00 (3.79) | 13.91 (3.45) |
| CSBS Consonant  Inventory | 3.87 (1.87) | 3.87 (2.00) | 3.88 (1.81) |  | 4.45 (2.39) | 4.09 (2.47) | 4.82 (2.36) |
| CSBS Comm. Acts with a Canonical Syllable | .28 (.21) | .27 (.20) | .29 (.22) |  | .32 (.19) | .29 (.20) | .35 (.18) |

*Note*. Elevated likelihood = Infants with at least one autistic older sibling, Population-level likelihood = Infants with only non-autistic older siblings. Looking to AV Synchrony = The proportion of time spent looking to the synchronous face relative to the total time spent looking to both faces. MSEL = Mullen Scales of Early Learning (Mullen, 1995); MSEL-ELC = Mullen Scales of Early Learning Early Learning Composite (Mullen, 2000; This standardized score is commonly used as a proxy for IQ); VABS = Vineland Adaptive Behavior Scales, Second Edition (Sparrow et al., 2005); MCDI = MacArthur-Bates Communicative Development Inventories, Words and Gestures (Fenson et al., 2007); AEQ = age equivalency scores; CSBS = Communication and Symbolic Behavior Scales Developmental Profile - Behavior Sample (Wetherby & Prizant, 2002); Comm. Acts = Communication acts.

**Table S2**

*Intercorrelations of Component Variables Used to Generate the Expressive Language and Vocalization Complexity Aggregates*

| Variable | 1 | 2 | 3 | 4 | 5 |
| --- | --- | --- | --- | --- | --- |
| 1. MSEL Expressive Language AEQ | — |  |  |  |  |
| 2. VABS Expressive Communication AEQ | .55^***^ | — |  |  |  |
| 3. MCDI Expressive Vocabulary | .61^***^ | .71^***^ | — |  |  |
| 4. CSBS Consonant Inventory | .34^*^ | .33^*^ | .43^**^ | — |  |
| 5. CSBS Comm. Acts with  a Canonical Syllable | .39^**^ | .38^**^ | .41^**^ | .57^***^ | — |

*Note*. MSEL = Mullen Scales of Early Learning (Mullen, 1995); VABS = Vineland Adaptive Behavior Scales, Second Edition (Sparrow et al., 2005); MCDI = MacArthur-Bates Communicative Development Inventories, Words and Gestures (Fenson et al., 2007); AEQ = age equivalency scores; CSBS = Communication and Symbolic Behavior Scales Developmental Profile - Behavior Sample (Wetherby & Prizant, 2002); Comm. Acts = Communication acts. Highlighted cells represent component variables that were aggregated together.

**p* < .05, ***p* < .01, ****p* < .001.
